# Personalized risk estimates of advanced neoplasia development in individuals with a family history of colorectal cancer

**DOI:** 10.1007/s10689-026-00565-0

**Published:** 2026-05-14

**Authors:** Marjolein J. E. Greuter, Eddymurphy U. Akwiwu, Simone D. Hennink, Tanya M. Bisseling, Marcel B. W. Spanier, German Consortium for Familial Intestinal Cancer, Iris D. Nagtegaal, Karen Canfell, Evelien Dekker, Thomas Klausch, Iris Lansdorp-Vogelaar, Monique E. van Leerdam, Nicoline Hoogerbrugge, Veerle M. H. Coupé

**Affiliations:** 1 Department of Epidemiology and Data Science, Amsterdam Public Health, Amsterdam UMC, Vrije Universiteit Amsterdam, C-371, Van der Boechorststraat 7, 1081 BT Amsterdam, The Netherlands; 2 Department of Gastroenterology and Hepatology, Medisch Spectrum Twente, Enschede, The Netherlands; 3 Department of Gastroenterology and Hepatology, Radboud University Medical Center, Nijmegen, The Netherlands; 4 Department of Gastroenterology and Hepatology, Rijnstate Hospital, Arnhem, The Netherlands; 5 Department of Pathology, Radboud University Medical Center, Radboud Institute for Molecular Life Sciences, Nijmegen, The Netherlands; 6 The Daffodil Centre, The University of Sydney, A Joint Venture with Cancer Council NSW, Sydney, Australia; 7 Department of Gastroenterology and Hepatology, Amsterdam University Medical Centers, University of Amsterdam, Amsterdam, The Netherlands; 8 Department of Public Health, Erasmus University Medical Center, Rotterdam, The Netherlands; 9 Department of Gastrointestinal Oncology, Netherlands Cancer Institute, Amsterdam, The Netherlands; 10 Department of Gastroenterology and Hepatology, Leiden University Medical Center, Leiden, The Netherlands; 11 Department of Human Genetics, Radboud University Medical Center, Research Institute for Medical Innovation, Nijmegen, The Netherlands

**Keywords:** Colorectal cancer, Adenoma, Dwell time, Familial cancer

## Abstract

To optimize surveillance in individuals with a family history (FH) of colorectal cancer (CRC), knowledge on the incidence rate of non-advanced adenomas (nAAs) and their progression rate to advanced neoplasia (AN) is crucial. We jointly estimated personalized adenoma incidence and progression rates using a novel statistical approach. We used data of individuals with ≥ 1 first-degree relative with CRC who underwent  ≥ 2 colonoscopies (*n* = 876 individuals; *n* = 2384 colonoscopies). Interval-censored data on timing and yield (no adenomas/nAA/AN) of each colonoscopy were available. nAA incidence and progression time from nAA to AN were estimated using a Bayesian progressive three-state model. Over a median follow-up of 6 years (interquartile range 5–6), 60 (6.8%) individuals developed AN (2 CRC, 58 AA) while 246 (28.1%) developed a nAA. The 5-year risk of developing nAA from baseline was estimated to be 29% (95% Crl: 21–39%). This risk was significantly associated with age and FH type. Estimated risk of progressing to AN within 5 years since nAA onset was 29% (95% Crl: 21–39%), and was significantly associated with having advanced adenomas at baseline. This resulted in a 5-year risk of transitioning from ‘no adenomas’ to AN of 7% (95% Crl: 5–9%). We showed that only 7% of individuals develop AN (primarily AAs) within the current 5-year colonoscopy surveillance interval. Future studies should evaluate whether this relatively low risk justifies slight extension of the surveillance interval or the use of fecal testing to guide colonoscopy timing. Furthermore, surveillance intensity could potentially be tailored based on age and FH.

**Supplementary Information:**

The online version contains supplementary material available at 10.1007/s10689-026-00565-0.

## Introduction

Colorectal cancer (CRC) is a prevalent disease. In 2022, there were almost two million CRC cases and one million CRC deaths worldwide [1]. Around 2–5% of CRCs are due to hereditary genetic disorders such as Lynch syndrome and familial adenomatous polyposis, which are caused by germline pathogenic variants in respectively one of the mismatch repair genes and the APC gene [2]. Another 30% of CRCs has a familial component without a known underlying genetic origin [2]. In these individuals with a family history (FH) of CRC, the cumulative lifetime risk of developing CRC is roughly 10% [3].

To reduce this risk, individuals with a FH of CRC are recommended surveillance with colonoscopy. During colonoscopy, precursor lesions, i.e. adenomas and serrated polyps, can be removed, thereby preventing CRC development. Note that the vast majority of CRCs arise from adenomas [4]. In addition, surveillance allows detection of CRC at an early stage, which leads to improved survival [5]. Although there is consensus that the increased CRC risk in this population justifies surveillance, there is no strong evidence-base to determine the optimal surveillance strategy. This is reflected by the differences in guideline recommendations. For example, the European Society of Gastrointestinal Endoscopy (ESGE) guideline [6] recommends five-yearly colonoscopy surveillance from age 40 onwards whereas the Australian guideline [7] recommends biennial stool-based testing between age 40–49 followed by five-yearly colonoscopy between age 50–74.

Ideally, the surveillance strategy would be informed by data-driven estimates on adenoma incidence rates and the duration from adenoma to advanced neoplasia (AN). However, estimating these rates is challenging as adenomas are removed upon detection. In addition, individuals with a FH of CRC represent a heterogeneous group, meaning that the CRC risk may vary considerably between individuals. These differences may be attributable to an individual’s characteristics (e.g. age and sex), type of FH (e.g. the number of first-degree relatives (FDRs) and the youngest age at which the FDR is diagnosed with CRC) and the yield at a previous colonoscopy (e.g. presence of an advanced adenoma (AA), defined as an adenoma with at least one of the following features: [1] ≥ 1 cm in size [2], with villous histology or [3] with high-grade dysplasia) [1, 3, 8, 9]. If these factors would impact adenoma incidence and progression rates, this would enable more personalised surveillance in which the surveillance strategy is tailored to an individual’s CRC risk. This study estimated adenoma incidence rates (i.e., the risk of developing non-advanced adenoma (nAA) from baseline) and the progression rates from onset of nAA to AN using a Bayesian accelerated failure time model for interval censored three-state surveillance outcomes [10]. Furthermore, we assessed which factors are associated with adenoma incidence and progression rates.

## Materials and methods

### Patient population and selection

For this study, we pooled prospective datasets of individuals who underwent surveillance colonoscopy because of a FH of CRC from three different sources: [1] data from the Dutch Familial Colorectal Cancer Surveillance (FACTS) randomized controlled trial [9, 2] observational data collected in the Dutch Radboudumc and Rijnstate hospitals, and [3] observational data from a German cohort. These three datasets are hereafter referred to as the FACTS, Radboudumc/Rijnstate and German cohorts, respectively. For the FACTS study, individuals with a FH of CRC were recruited by informing all gastroenterologists and GPs in the Netherlands about the FACTS study with an information package [11]. The Radboudumc/Rijnstate cohort and the German cohort consist of individuals who were suspected of having Lynch syndrome, based on the Amsterdam and Bethesda criteria. The family history was verified with medical and pathology reports. In suspected cases of hereditary CRC syndrome (e.g. CRC diagnosed at age < 50 years) tumors were analyzed for microsatellite instability (MSI), and patients with MSI-high tumors were referred to clinical genetic centers for further evaluation, including germline mutation analysis of mismatch repair genes (MLH1, MSH2, MSH6, PMS2, and EPCAM). Families with confirmed Lynch syndrome were excluded from the cohort. Of these cohorts, we selected individuals who fell in one of the following FH categories, that is, (a) 1 FDR diagnosed with CRC before the age of 50, (b) at least 2 FDRs diagnosed with CRC between age 50–70 and (c) at least 2 FDRs diagnosed with CRC of which at least one was diagnosed before the age of 50. In addition, we only included individuals who underwent at least two complete colonoscopies. Of each colonoscopy, information on the date and yield had to be available. For the purpose of this study, each individual’s first colonoscopy reported in the dataset was considered the baseline examination, with its outcome (i.e., presence or absence of adenoma) included in the model (see Section “Estimation method and data analysis”). Note that the first colonoscopy in the dataset is not necessarily the first colonoscopy in the individual’s lifetime. We excluded individuals who were previously diagnosed with CRC or had CRC at baseline colonoscopy (Flowchart, Supplemental 1).

### Multi-state model

**Fig. 1:**
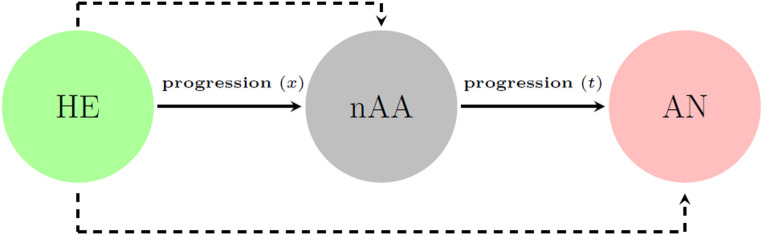
A progressive three-state model depicting the natural history of advanced neoplasia (AN) development with health states: healthy (HE), non-advanced adenoma (nAA) and AN. HE includes individuals without adenomas and those with adenomas at baseline that are completely removed by polypectomy. Underlying process (solid lines) and observed process (dashed lines)

Since adenoma incidence and progression rates cannot be directly observed, as the true underlying (latent) process is only observed at a limited number of discrete points in time, we estimated these rates by describing the multi-state development of AN using a progressive three-state disease model (Fig. 1). The health states include healthy (HE), nAA and AN (defined as AA or CRC). We denote *x* and *t* as the progression times from HE to nAA and from nAA onset to AN, respectively. Here, nAA onset refers to the moment at which an nAA develops in the natural history of the disease, that is, the initiation of nAA in the absence of surveillance. We assume that all individuals with adenomas of any size are successfully treated by means of polypectomy at baseline. Consequently, individuals with adenomas at baseline are classified as HE after removal, and as such everyone is considered to be in the HE state at baseline. In the assumed underlying disease process (i.e., in the absence of surveillance), which is based on the adenoma-carcinoma sequence [12], individuals progress from HE to nAA and subsequently from nAA onset to AN. Note that the transition from nAA onset to AN is not observed in clinical practice because nAAs are removed upon detection. AN is only observed in individuals who made both the transition from HE to nAA as well as the transition from nAA onset to AN between two colonoscopy visits. This information is used to estimate the progression rate from nAA onset to AN while accounting for the transition time from HE to nAA. In addition, we assume that the surveillance test is perfect, that is, its sensitivity and specificity are 100%. Details of the observation process during surveillance that describes how individuals are classified into the three states, are provided in Supplemental 2.

### Estimation method and data analysis

To estimate the progression times (*x*, *t*) from HE to nAA and from nAA to AN based on the observation process described above, we fitted a multivariable model using the BayesTSM (Bayesian three-state model) R software package [10] and assessed which variables are associated with the hazard of developing nAA from baseline and the hazard of developing AN after nAA onset, with both hazards modelled using a Weibull distribution. The variables assessed were: age at baseline, sex, type of FH and the presence of an AA at baseline (yes/no). Including presence or absence of AA at baseline as a covariate allowed us to evaluate whether the nAA incidence and progression rates differed between individuals with and without an AA removed at baseline. We computed the hazard ratios (HRs) and the corresponding 95% credible intervals (Crls) for each variable in the model. We considered a variable statistically significant if 1 is not included in the 95% Crl of the HR. We estimated the cumulative probability of developing an event (nAA or AN) for various time points, averaged over all individuals in the population (i.e., marginal cumulative incidence function (CIF)), thus indicating the population-average risk [13]. In addition, given evidence of substantial heterogeneity in progression in the literature [14], characterized by early progression for some lesions and very long transition times (i.e., slow or no progression) for others, we estimated the mean transition time conditional on progression within a prespecified follow-up period (e.g., 20 years) to provide an estimate of the average time to progression among individuals who develop the event within that period. To give insight into the differences in risk across levels of the significant covariates for both transitions, we estimated the conditional CIFs up to 10 years after baseline. The conditional CIF is the cumulative probability of developing an event at a specific time point for a specific covariate profile. As all variables in the model need to be specified to estimate the conditional CIFs, we created a ‘high’ and ‘low’ risk profile. We defined the high- and low-risk profiles by setting all variables to the levels corresponding to the highest and lowest risk, respectively (details in Supplemental 3). Plots of conditional CIFs stratified by levels of the significant variables in the model were produced for both the ‘high-’ and ‘low-risk’ profiles.

We conducted two additional analyses (details in Supplemental 3). In the first analysis, all analyses were repeated using only the Dutch population instead of the pooled Dutch and German populations (i.e., base-case analysis). Furthermore, while the base-case analysis assumed perfect sensitivity for all colonoscopies (both baseline and follow-up). Although this is a reasonable assumption for advanced neoplasia, it is optimistic with regard to the detection of nAAs [15]. Therefore, we performed an exploratory analysis using a preliminary extended model, in which the sensitivity for detecting nAA and AN during follow-up was set at 75% and 100%, respectively. Note that this preliminary model assumes perfect sensitivity for the baseline colonoscopy. All analyses were conducted using statistical software R, version 4.1.2 [16].

## Results

### Participant’s characteristics

Table 1 shows the baseline characteristics of the total population (*n* = 876 individuals; *n* = 2384 colonoscopies). The average age was 51.5 years. The majority did not have an AA at the baseline colonoscopy (95.8%). Note that the German cohort was on average younger than the Dutch population (*P* < 0.001) and had a lower proportion of individuals with AA detected at baseline colonoscopy (*P* = 0.043). Furthermore, the FH groups were significantly different between the Dutch and German cohorts (overall *P* < 0.001). There were considerably more individuals with at least 2 FDRs diagnosed with CRC of which at least one was diagnosed before the age of 50 in the German cohort (60.6%) compared to the Dutch cohort (15.0%). Median follow-up (interquartile range) was 6 (5–6) years. The distribution of the number of surveillance colonoscopies per participant is reported in Supplemental 4, with a median of 3 (interquartile range 2–4). The findings at the time of censoring, that is, first detection of a nAA or AN, or HE in case the end of follow-up is reached (or due to loss to follow-up) without detection of nAA or AN, is also shown in Table 1. In the total population, 60 (6.8%) individuals had an AN while 246 (28.1%) had a nAA and 570 (65.1%) individuals remained in the HE state until the last date of follow-up visit. These proportions were similar for the Dutch population with 6.7%, 28.3% and 65.0%, respectively.

**Table 1:** Baseline characteristics of the study population and distribution of findings at the time of censoring

	Total population	Dutch population	German population
	(*n* = 876)	(*n* = 734)	(*n* = 142)
*Participant characteristic*			
Age at baseline, yr, mean (SD)	51.5 (9.1)	53.0 (7.4)	43.4 (12.3)
*Sex, n (%)*			
Male	374 (42.7)	327 (44.6)	47 (33.1)
Female	502 (57.3)	407 (55.4)	95 (66.9)
*Advanced adenoma at baseline, n (%)*			
Yes	37 (4.2)	33 (4.5)	4 (2.8)
No^a^	839 (95.8)	701 (95.5)	138 (97.2)
*Study, n (%)*			
Radboud/Rijnstate	227 (25.9)	227 (30.9)	–
FACTS	507 (57.9)	507 (69.1)	–
German	142 (16.2)	–	142 (100)
*Family history, n (%)*			
1 FDR with CRC < 50	404 (46.1)	358 (48.8)	46 (32.4)
*≥* 2 FDRs with CRC 50–70	276 (31.5)	266 (36.2)	10 (7.0)
*≥* 2 FDRs with *≥* 1 CRC < 50	196 (22.4)	110 (15.0)	86 (60.6)
*Findings at the time of censoring* ^b^			
Healthy, n (%)	570 (65.1)	477 (65.0)	93 (65.5)
Non-advanced adenoma, n (%)	246 (28.1)	208 (28.3)	38 (26.8)
Advanced neoplasia, n (%)	60 (6.8)	49 (6.7)	11 (7.7)

FDR, First-degree relative

^a^Includes no finding + non-advanced adenoma

^b^An individual is censored during follow-up once the first non-advanced adenoma or advanced neoplasia is detected, or at the end of follow-up period when they remained healthy during follow-up

### Adenoma incidence and progression from nAA to AN

**Fig. 2:**
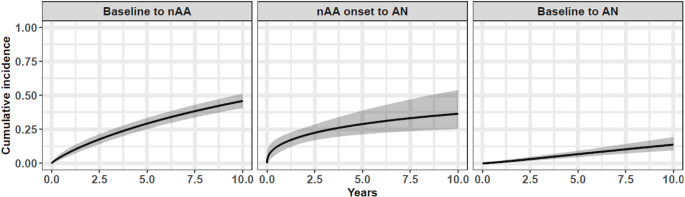
Estimated marginal cumulative incidence functions representing the population-average risks to develop nAA from baseline, AN after nAA onset and AN from baseline based on the total population. The cumulative incidence of AN from baseline to an arbitrary timepoint (right panel) is obtained by calculating the proportion of combined event times from baseline to nAA and nAA to AN (from the time distributions from the left and middle panels) that give a sum less than or equal to this given timepoint. Estimates are based on the full multivariate model (Supplemental Table S2). The black solid line represents the average estimate while the grey shaded area represents the 95% Crl. Note that all individuals are included in the modelled curve, rather than depicting the cumulative incidence for a specific covariate profile

Figure 2 (left and middle panels) depicts the marginal cumulative incidence curves for both transition times (i.e., HE to nAA and nAA to AN), showing a decreasing overall hazard over time for both transitions (see also Supplemental 5). As mentioned earlier, the term ‘marginal’ implies that the modelled curve represents an average rather than depicting the cumulative incidence for a specific covariate profile. The 5-year and 10-year population-average risks of developing nAA from baseline were estimated to be 29.2% (95% Crl: 25.2–33.6%) and 45.9% (95% Crl: 40.5–51.1%), respectively. Among individuals who developed nAA within 20 years, the mean time to nAA was 8.2 years (95% Crl: 7.1–9.2). The corresponding estimates for the risk of developing AN since nAA onset were 29.1% (95% Crl: 21.3–38.8%) and 36.8% (95% Crl: 25.5−53.4%), respectively, with a mean time to AN of 3.4 years (95% Crl: 0.7–6.0) among those progressing within 20 years. The right panel in Figure  2 depicts the sum of the two transitions. This curve shows that only 6.7% (95% Crl: 4.6–9.2%) and 13.8% (95% Crl: 9.6–19.3%) of individuals will develop AN within 5 and 10 years from baseline, respectively, with a mean time of 9.6 years (95% Crl: 8.2–11.0) among those progressing from the healthy state to AN via nAA within 20 years.

Individuals with at least 2 FDRs diagnosed with CRC of which at least one was diagnosed before the age of 50 showed a significantly higher hazard of developing nAA from baseline compared to those with one FDR diagnosed with CRC before the age of 50 years (HR = 1.36, 95% Crl: 1.01–1.83), but this was not the case for the group with at least two FDRs diagnosed with CRC between the ages of 50–70 years (Table 2). Similarly, baseline age was significantly associated with the hazard of developing nAA from baseline, with each additional year of age increasing the risk by 2% (HR = 1.02, 95% Crl: 1.01–1.04). This gives a 10-year HR of 1.25 (95% Crl: 1.09–1.44); translating to a 25% increase in the hazard of developing nAA per 10-year increase in age. There were no significant associations between the other covariates and the hazard to develop nAA from baseline. In contrast, only the presence of AA removed at baseline was significantly associated with the hazard of progression from nAA to AN (HR = 2.20, 95% Crl: 1.01–5.62).

**Table 2:** Hazard ratios showing the association between each covariate and the hazard of developing nAA from baseline and the hazard of developing AN since nAA onset in the total population with 876 individuals

Transition	Distribution	Covariate	HR	95% Crl
Baseline ⟶ nAA	Weibull	$\:\ge\:2$ FDRs with CRC 50–70^a^	1.16	(0.89, 1.53)
		$\:\ge\:2$ FDRs with $\:\ge\:1$ CRC < 50 ^a^	**1.36**	**(1.01**,** 1.83)**
		Sex: Male	1.14	(0.91, 1.42)
		Age	**1.02**	**(1.01**,** 1.04)**
		AA: Yes	1.37	(0.84, 2.15)
		Study: German ^b^	1.20	(0.85, 1.67)
		$\:\ge\:2$ FDRs with CRC 50–70^a^	1.11	(0.70, 1.85)
nAA ⟶ AN	Weibull	$\:\ge\:2$ FDRs with $\:\ge\:1$ CRC < 50 ^a^	0.81	(0.44, 1.34)
		Sex: Male	1.38	(0.88, 2.26)
		Age	1.00	(0.97, 1.03)
		AA: Yes	**2.20**	**(1.01**,** 5.62)**
		Study: German ^b^	1.53	(0.83, 3.62)

nAA, Non-advanced adenoma; AA, Advanced adenoma; AN, Advanced neoplasia; CRC, Colorectal cancer; HR, Hazard ratio; 95% Crl, 95% credible interval; FDR, First-degree relative

^a^Reference group is 1 FDR with CRC < 50

^b^Included as an additional covariate in the model (i.e., a dummy variable) to account for possible cohort effect due to combining data from Dutch and German cohorts; similar to the approach by Lange et al. [17] Values in bold font indicate hazard ratios for which the 95% credible interval does not include 1 and are therefore considered statistically significant.

**Fig. 3:**
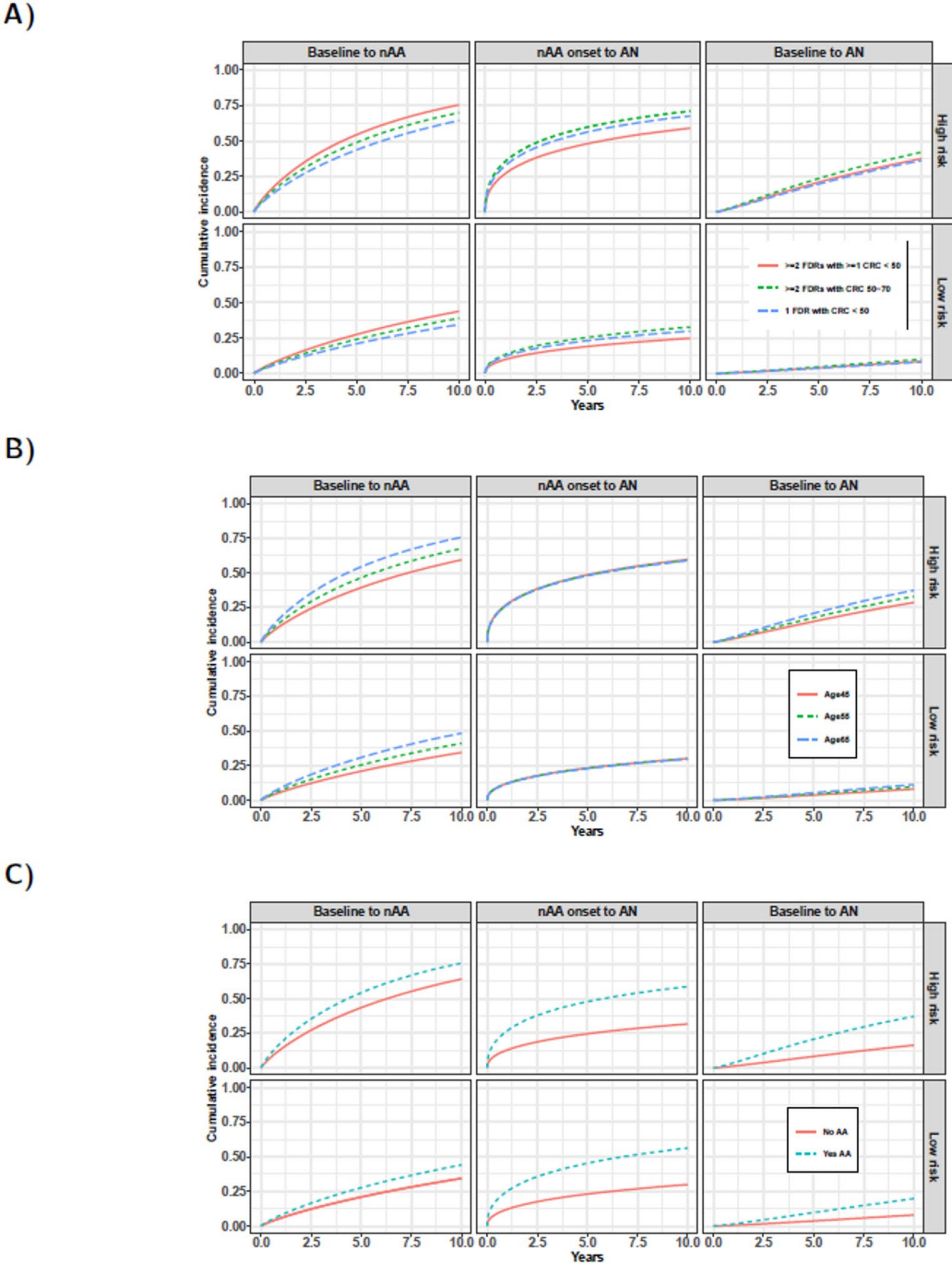
Conditional cumulative incidence functions representing the individual-specific risks to develop nAA from baseline, AN after nAA onset and AN from baseline based on the total population. Estimates for ‘High-’ and ‘Low-risk’ individuals were stratified by **a** the different family history groups; **b** different baseline ages (45, 55, 65 years); and the **c** presence of AA removed at baseline (yes/no). Note that estimates were based on the full multivariate model (Supplemental Table S2) and all other covariates were therefore based on a high- and low-risk profile. ‘High-risk‘ individuals were defined as Dutch males aged 65 years with at least 2 FDRs diagnosed with CRC of which at least one was diagnosed before the age of 50. ‘Low-risk’ individuals were defined as Dutch females aged 45 years with 1 FDR who was diagnosed with CRC before age 50 years and with no AA removed at baseline

Based on these analyses, the high-risk profile was defined as males aged 65 years with at least 2 FDRs diagnosed with CRC, of which at least one was diagnosed before the age of 50, and with AA removed at baseline. Female individuals aged 45 years with 1 FDR who was diagnosed with CRC before age 50 years and with no AA removed at baseline were simulated for the low-risk profile. Figure 3 displays the conditional cumulative incidence of developing nAA from baseline and AN since nAA onset for the different FH groups, age and presence of an AA removed at baseline for the ‘high-’ and ‘low-risk’ profile (with respect to the remaining covariates). Both for the high- as well as the low-risk profile, individuals with at least 2 FDRs diagnosed with CRC of which at least one was diagnosed before the age of 50, had the highest nAA risk. Conversely, individuals with 1 FDR diagnosed with CRC before the age of 50 had the lowest nAA risk. For example, the 5-year risk of developing a nAA from baseline for Dutch males aged 65 years with at least 2 FDRs diagnosed with CRC of which at least one was diagnosed before the age of 50 and with AA removed at baseline, was estimated to be 54.5% (95% Crl: 36.7%–72.2%) as compared to 43.8% (95% Crl: 28.1%–60.9%) for individuals with the same risk profile but with 1 FDR diagnosed with CRC before the age of 50. Similar differences in risk were observed between the FH groups for the low-risk profile.

For age, the 5-year risk of developing nAA from baseline for ‘high-risk’ individuals aged 45, 55 and 65 years was respectively 39.2% (95% Crl: 25.3–56.5%), 46.7% (95% Crl: 30.9–63.4%) and 54.4% (95% Crl: 36.6–72.2%). ‘Low-risk’ individuals showed a similar increase in risk with baseline age. For the presence of an AA at baseline, the 5-year risk of developing AN since nAA onset for ‘high-risk’ individuals with AA removed at baseline was estimated to be 47.4% (95% Crl: 20.1–85.2%) as compared to 24.6% (95% Crl: 12.6–43.2%) for individuals with the same risk profile but with no AA removed at baseline. A similar difference in risks was observed between these two groups of individuals (AA vs. no AA) for the low-risk profile. Note that the risks of developing AN since nAA onset for individuals with AA removed at baseline and those with no AA removed at baseline (middle panel, Fig. 3c), were similar for both the ‘high-risk’ and ‘low-risk’ profiles. This similarity is due to the fact that none of the other covariates were statistically significantly associated with the hazard of developing AN since nAA onset (Table 2).

The results of the additional analysis using the Dutch cohorts were similar to the base-case analysis, except that none of the family history groups were significantly associated with the hazard of developing nAA from baseline (Supplemental 6). This is likely due to the smaller sample size (*n* = 734 for the Dutch population and *n* = 876 for the total population). Additionally, the exploratory analysis, in which we assumed imperfect sensitivity of follow-up colonoscopies for nAA, showed differences in population-average risks (Supplemental 7). For example, the 5-year risk of developing nAA from baseline was 29.2% (95% Crl: 25.2%–33.6%) in the base-case vs. 35.2% (95% Crl: 30.7–40.0%) in the exploratory analysis. For AN development after nAA onset, the 5-year risk was 29.1% (95% Crl: 21.3–38.8%) vs. 22.7% (95% Crl: 16.9–29.9%), respectively. The corresponding estimates for the risk of developing AN from baseline were similar between the base-case (6.7%, 95 Crl: 4.6%–9.2%) and the exploratory analysis (6.6%, 95% Crl: 4.5–9.0%).

## Discussion

This study provides insight in the natural history of advanced neoplasia (AN) development in individuals with a FH of colorectal cancer (CRC) in order to guide evidence-based recommendations for the optimal surveillance strategy. The 5-year and 10-year population-average risks of developing a non-advanced adenoma (nAA) from baseline, i.e. a negative colonoscopy or a colonoscopy during which all lesions were removed, were estimated to be 29% and 46%, respectively. The risk of developing nAA from baseline was significantly associated with age (10-year HR: 1.25) and type of FH (≥ 2 FDRs with ≥ 1 CRC < 50 compared with 1 FDR with CRC < 50: HR 1.36). The 5-year and 10-year population-average risks of developing AN since nAA onset were 29% and 37%. The risk of developing AN was significantly associated with presence of an advanced adenoma (AA) at baseline (HR: 2.20). This resulted in an average 5-year and 10-year risk of transitioning from ‘no adenomas’ to AN of 7% and 14%, respectively.

Although knowledge on adenoma incidence and progression rates is crucial to determine the optimal surveillance strategy, they are challenging to estimate since adenomas are removed upon detection. Combined with the interval-censored nature of surveillance data, this necessitates sophisticated statistical methods, such as the Bayesian three-state model that we used. To our knowledge, this is the first study that estimated adenoma incidence and progression rates in individuals with a FH of CRC. Previously, Brenner et al. [18] estimated adenoma incidence rates in average-risk individuals. Similar to our results, they showed that men have a higher risk than women. However, in contrast to our study, this difference was significant. This difference could potentially be explained by their large sample size (4.3 million individuals). Interestingly, they found similar adenoma incidence across ages whereas we observed a significant increase with age. Subsequently, Brenner et al. [18] estimated 10-year adenoma and AA risks in the average-risk population. The 10-year adenoma risk for men and women aged 55 after a negative colonoscopy was respectively 20% and 13% whereas the 10-year AA risk was respectively 3% and 2%. We found a 46% population-average risk of developing an adenoma within 10 years since baseline in individuals with a FH of CRC. This risk was 14% for the transition from healthy to advanced neoplasia, most of which were advanced adenomas. As expected, our risks are considerably higher. This may be due to including individuals with (advanced) adenomas at baseline colonoscopy. Still, even for our low-risk profile (female aged 45 years with 1 FDR who was diagnosed with CRC before age 50 years and with no AA removed at baseline) with 10-year adenoma and advanced neoplasia risks of 34% and 8%, respectively (cf. Figure 3), our 10-year risk estimates were markedly higher. Therefore, the most likely reason is our focus on an increased-risk population whereas Brenner et al. [18] focused on an average-risk population. This justifies the more intensive surveillance in this population.

While it is well established that a FH increases CRC risk [3], there is limited evidence whether this increased risk is caused by a higher adenoma incidence and/or by faster progression from adenoma to CRC. A recent modelling study showed that it is likely that individuals with a FH have a higher adenoma incidence [19]. Our findings support this, as our estimated adenoma incidence rates are roughly three times higher than Brenner et al.’s estimates [18] (10-year risk 46% vs. 16.5%). This increased incidence may potentially be combined with faster progression, as the 10-year risk from healthy to AN was roughly 5.5 times higher in individuals with a FH of CRC compared to the risks reported by Brenner et al. [18] (10-year risk 14% vs. 2.5%). Applying the Bayesian three-state model to an average-risk population could clarify the mechanism that causes the increased risk.

The current ESGE surveillance guideline recommends 5-yearly surveillance in this increased risk population [6]. We showed that the risk of developing AN, of which most were AAs, within this surveillance interval is relatively low (7%). This suggests surveillance may be too intensive, at least in some subgroups, leading to unnecessary burden. Slight extension of the 5-year surveillance interval might be acceptable, as also indicated by the Familial Colorectal Cancer Surveillance study [9]. This randomized controlled trial compared a 3-yearly with a 6-yearly surveillance interval. After 6 years of follow-up, there was no significant difference in the proportion of patients with AA. More research is needed to determine to what extent the surveillance interval could be safely extended in individuals with a FH of CRC. Even more promising might be extension of the colonoscopy surveillance interval combined with non-invasive fecal testing to guide colonoscopy timing. Indeed, a modelling study found that the optimal surveillance strategy in this population would be ten-yearly colonoscopy combined with two-yearly fecal testing in between [19]. Such a strategy was more effective at lower costs and would considerably reduce the number of colonoscopies that individuals should undergo. An ongoing randomized controlled trial will clarify whether fecal testing can be used to extend the colonoscopy surveillance interval in individuals with a FH [20]. Given the long follow-up period required, results are not anticipated in the near future. Importantly, any change in surveillance interval or strategy will require broad support among clinicians, and should be based on a comprehensive evaluation of effectiveness, cost-effectiveness, and acceptability to both patients and healthcare providers.

We observed a decreasing hazard over time for both nAA development and the progression from nAA to AN. As suggested previously [14], this reduction in hazard may be attributed to heterogeneity within the population. This heterogeneity reflects differences among individuals, including those who progress rapidly to the next disease state and those who progress more slowly or not at all. Additionally, our findings indicate that the risk of developing nAA increases with age. These two mechanisms—age-related effects on disease progression and the selection process, where lower-risk individuals increasingly remain in the risk set over time—interact. For instance, while an individual’s risk of developing nAA in the next five years increases with age, this same individual’s overall risk decreases over time if they have remained disease-free. This heterogeneity is further demonstrated, for example, by the short mean time to progression from nAA to AN among those progressing within 20 years (3.4 years) and indicates the need for personalized surveillance.

To enable personalized surveillance, in which the surveillance interval is tailored to an individual’s CRC risk, it is important to identify factors associated with the heterogeneity in risk. Indeed, we showed that age and FH are associated with nAA development. Several meta-analyses also indicated that CRC risk differs based on type of FH [3, 21] but did not perform statistical tests to directly compare the groups. We showed that the risk of developing adenomas differs significantly between individuals with ≥ 2 FDRs with CRC of which one is diagnosed before the age of 50 compared to individuals with 1 FDR with CRC diagnosed before the age of 50. Future studies should evaluate whether this difference in risk is substantial enough to personalize surveillance based on FH and age. Furthermore, we found that the presence of an AA at a previous colonoscopy is associated with nAA to AN progression. The presence of an AA is a well-known risk factor and therefore, those individuals are advised to undergo more intensive surveillance according to the post-polypectomy guideline, i.e. post-polypectomy surveillance [22, 23]. Note that this surveillance differs from the standard surveillance for individuals with a FH of CRC.

This work is not without limitations. First, we assumed that all colonoscopies, i.e. baseline colonoscopy and follow-up colonoscopies, have perfect sensitivity. In an exploratory analysis, we assessed the impact of this assumption by accounting for imperfect sensitivity in follow-up colonoscopies. Note that this is an approximation of the true impact of imperfect sensitivity as also the baseline colonoscopy has imperfect sensitivity. This means that in reality not all individuals will be adenoma-free after colonoscopy, while we assume that that is the case. This exploratory analysis showed that we slightly underestimated the adenoma incidence rate and slightly overestimated the progression rate from nAA to AN. Estimates for the transition from healthy to AN were comparable. Imperfect sensitivity at baseline is currently added to the BayesTSM package. Second, due to the effectiveness of colonoscopy surveillance, only two individuals in our dataset developed CRC during follow-up. Therefore, we could not estimate the time from AA to CRC. Considerably larger surveillance datasets are required to enable this. Furthermore, we assumed that all CRCs developed from adenomas while also serrated lesions can progress to CRC [24]. As the majority of CRCs develop via the adenoma-carcinoma pathway and we had few cancers in our dataset, we are fairly certain that this would not have impacted our results. Estimating incidence and progression rates for serrated polyps would also be interesting, but this would also require large datasets. Finally, we did not take the multiplicity of adenomas into account. This could also have an impact on our risk estimates.

To conclude, we showed that only 7% of individuals transition to advanced neoplasia (mostly AAs) within the current 5-year colonoscopy surveillance interval. Future studies should evaluate whether this relatively low risk justifies slight extension of the current 5-year surveillance interval or the use of fecal testing to guide colonoscopy timing. In addition, surveillance intensity could potentially be tailored based on age and of type of FH.

## Supplementary Information

Below is the link to the electronic supplementary material.

Supplementary Material 1

## Data Availability

The data is available upon reasonable request.
