# Supplementary material for: Personalized risk estimates of advanced neoplasia development in individuals with a family history of colorectal cancer
Source: Fam Cancer. 2026 May 14;25(2):56. doi: 10.1007/s10689-026-00565-0 (PMC13175997; doi:10.1007/s10689-026-00565-0)
Supplement: Supplementary file 1 — Supplementary Material 1 [file 10689_2026_565_MOESM1_ESM.pdf]

Supplementary material for: Personalized risk estimates of advanced neoplasia development in individuals with a family history of colorectal cancer

Contents

1 Study flowchart of participants with family history of CRC 2

2 Observation process during surveillance 3

3 Additional details of the data analysis 5

4 Distribution of the number of surveillance colonoscopies 6

5 Additional results from the multivariate analysis based on the total population (i.e. base-case analysis) 7

6 Additional results from the multivariate analysis based on the Dutch population only (i.e. sensitivity analysis) 8

7 Additional results comparing perfect sensitivity and imperfect sensitivity of colonoscopy in detecting nAAs (i.e. exploratory analysis) 11

1 Study flowchart of participants with family history of CRC

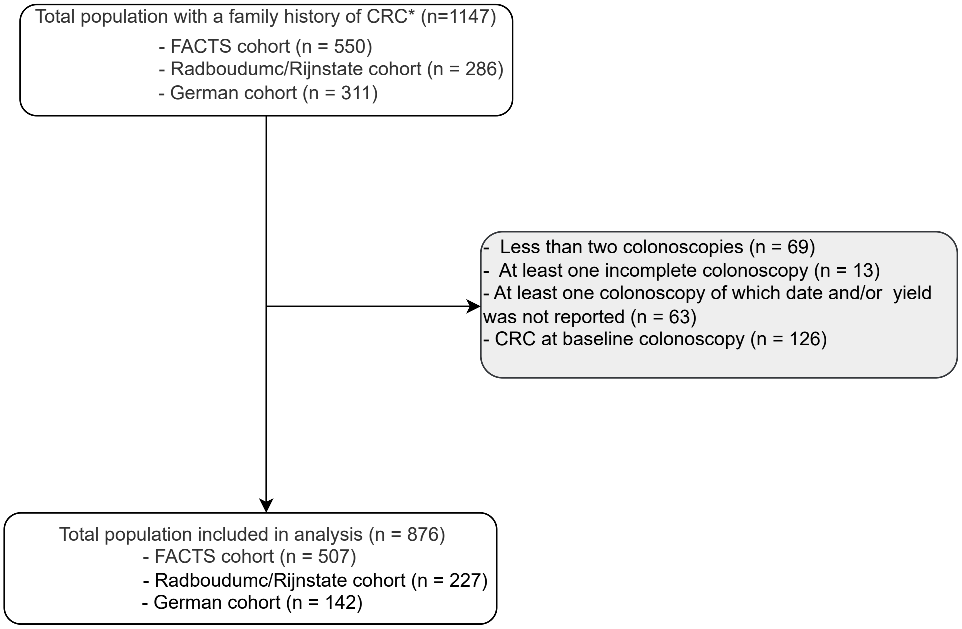

Figure S1. Study flowchart of participants with family history of CRC.
*A family history of CRC was defined as: (1) 1 FDR diagnosed with CRC <50 years, (2) At least 2 FDRs diagnosed with CRC between 50-70 years and (3) At least 2 FDRs diagnosed with CRC of which at least one is diagnosed <50 years.

2 Observation process during surveillance

During surveillance, individuals are followed up with colonoscopy, according to a predefined schedule, say $\boldsymbol{v} = (v_1, v_2, \cdots, v_{m-1}, v_m)$, which may or may not be strictly adhered to in terms of interval between colonoscopies. This means that the schedule, i.e., number of colonoscopies m and the interval between colonoscopies is allowed to vary across individuals. We assume that the surveillance test is perfect, that is, its sensitivity and specificity are 100%. Although this is a reasonable assumption for advanced neoplasia, it is optimistic with regard to the detection of non-advanced adenomas (nAAs) (1). For the purpose of this study, we are only interested in the time of first detection of a nAA or an advanced neoplasia (AN) after baseline, at which point the individual is treated (i.e., the adenoma is removed) and censored, as well as the last date of follow-up visit (either due to loss to follow-up or end of follow-up period) for individuals in whom neither a nAA nor an AN was detected during follow-up. The observation process is illustrated through three typical scenarios that may occur during surveillance (Figure S2). In the first scenario, neither nAA nor AN is observed within the follow-up period, and therefore, if it occurs, it happens after the last visit v_m. This leads to right-censoring. In the second scenario, nAA is observed during follow-up at visit v_m and is immediately treated, after which the individual is censored. In the third scenario, AN is observed during follow-up at visit v_m, which necessarily implies that the transition from healthy (HE) to nAA occurred within the same surveillance interval. The second and third scenarios lead to interval-censoring since the actual time of development of nAA or AN is not precisely observed but is only known to lie between two consecutive visit times, $(v_{m-1}, v_m]$. Therefore, the finding at the time of censoring for an individual during follow-up will be either HE (first scenario), nAA (second scenario), or AN (third scenario).

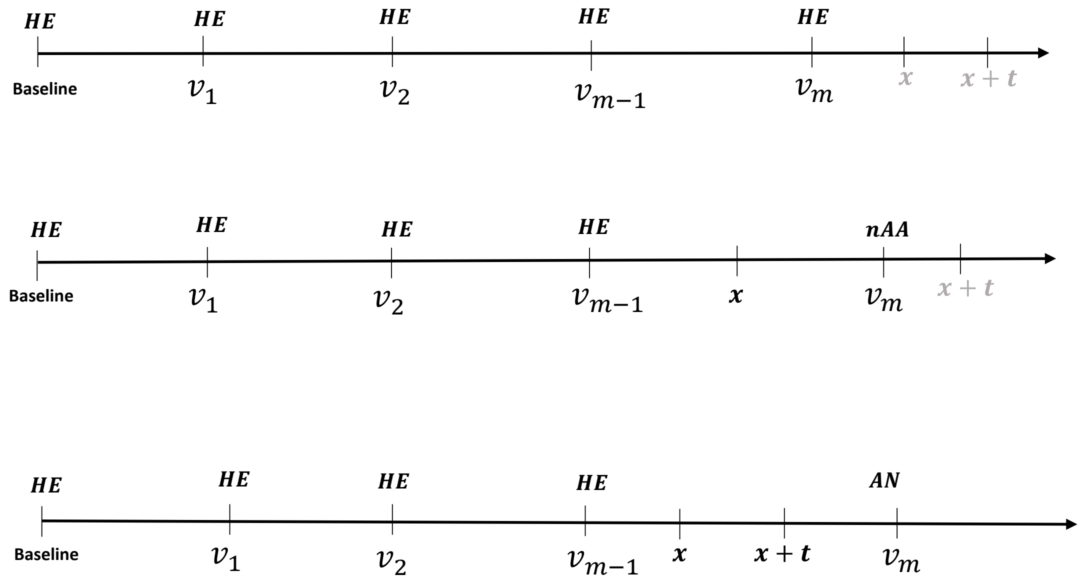

Figure S2. Three typical scenarios during surveillance where an individual could either (i) remain HE (top panel) up till the end of follow-up v_m, (ii) be detected with a nAA (middle panel) at v_m with the nAA occurring between $(v_{m-1}, v_m]$, or (iii) be detected with an AN (bottom panel) at v_m with the AN occurring between $(v_{m-1}, v_m]$ during follow-up. HE includes individuals without adenomas and those with adenomas at baseline after complete removal by polypectomy.

3 Additional details of the data analysis

We fitted a multivariable model to assess which variables are associated with the hazard of developing nAA from baseline and the hazard of developing AN after nAA onset. The variables assessed were: age at baseline, sex, type of family history and the presence of an AA at baseline (yes/no). Similar to Lange et al. (2), we accounted for a possible cohort effect due to combining data from Dutch and German cohorts, which vary in baseline characteristics, by including a dummy variable for the country of study (Dutch/German) as an additional covariate in the model.

Parameter estimates from the `BayesTSM` (Bayesian three-state model) `R` package were used to compute the marginal and conditional cumulative incidence functions (CIFs) of nAA and AN. To give insight into the differences in risk across levels of the significant covariates for both transitions (i.e., HE to nAA and nAA to AN), we estimated the conditional CIFs up to 10 years after baseline. As all variables in the model need to be specified to estimate the conditional CIFs, we created a 'high' and 'low' risk profile. Plots of conditional CIFs stratified by levels of the significant variables in the model were produced for both the 'high-' and 'low-risk' profiles. We defined the high- and low-risk profiles by setting all variables to the levels corresponding to the highest and lowest risk, respectively. For example, if men were found to be at higher risk than women, a male population would be simulated for the high-risk profile, while a female population would be simulated for the low-risk profile. As age was included as a continuous variable in the model, we used age 45 for the low-risk profile and age 65 for the high-risk profile, as these ages are below and above the average age in the data (i.e., age 52 years).

In the base-case analysis, the Dutch and German studies are pooled. Although we accounted for a possible cohort effect due to differences in baseline characteristics by including a dummy variable for country of study, we further assessed the potential impact of combining these populations in a sensitivity analysis. In this analysis, we repeated all analyses using the Dutch population only. Unfortunately, it was not possible to repeat the analyses in the German population only due to the small sample size ($n = 142$). In addition, we assumed in our base-case analysis that all colonoscopies, i.e. both the baseline and follow-up colonoscopies, have perfect sensitivity as the three-state model currently does not allow for analyses assuming imperfect sensitivity. Although this assumption is reasonable for AA, it is optimistic for nAA as literature shows that colonoscopies miss around 25% of nAAs (1). To get an impression of the impact of imperfect sensitivity on the cumulative incidence functions of developing nAA from baseline and AN development since nAA onset, we conducted an exploratory analysis with a preliminary extended model in which we set the sensitivity of detecting nAA and AN during follow-up at 75% and 100%, respectively. This preliminary model only accounts for imperfect sensitivity for follow-up colonoscopies, but not for the baseline colonoscopy. Thus, although nAA can be missed during follow-up, the model still assumes that all individuals start as healthy, while in reality there could be nAA missed at baseline.

4 Distribution of the number of surveillance colonoscopies

Table S1. Distribution of the number of surveillance colonoscopies per participant in the total population.

**Number of participants**	477	294	51	25	10	9	2	4	2	1	1
**Number of colonoscopies**	2	3	4	5	6	7	8	9	10	11	13

5 Additional results from the multivariate analysis based on the total population (i.e. base-case analysis)

Table S2 displays the parameter estimates of the full model, including the Weibull shape parameters. The Weibull shape parameters for the progression times from HE to nAA and from nAA to AN were estimated to be 0.84 (95% CrI: 0.71–0.98) and 0.43 (95% CrI: 0.21–0.77), respectively. These estimates translate to a decreasing hazard to develop nAA from baseline and a decreasing hazard to develop AN since the onset of nAA, respectively. In other words, the hazard of developing a nAA from a healthy state decreases for every additional year that an individual remains HE. Similarly, for the progression from nAA to AN, the risk decreases for every additional year that an individual has the nAA. These estimates indicate a decreasing overall hazard over time for both transitions. In other words, the longer an individual remains in a healthy state without developing nAA, the less likely they are to develop nAA in the future. Similarly, for individuals who have already developed nAA, the risk of progressing to AN decreases with each additional year they remain in the nAA state. This trend is also illustrated in the marginal cumulative incidence curves (left and middle panels) in Figure 2 of the main manuscript.

Table S2. Result of the multivariate analysis showing the parameter estimates of the full model with $n = 876$ individuals.

Transition	Distribution	Covariate	Estimate	95% CrI
Baseline → nAA	Weibull	Shape	0.84	(0.71, 0.98)
		Intercept	3.15	(2.87, 3.51)
		≥ 2 FDRs with CRC 50-70†	0.16	(-0.12, 0.43)
		≥ 2 FDRs with ≥ 1 CRC <50†	**0.31**	**(0.01, 0.61)**
		Sex:Male	0.13	(-0.10, 0.35)
		Age	**0.02**	**(0.01, 0.04)**
		AA:Yes	0.32	(-0.17, 0.76)
		Study:German§	0.18	(-0.16, 0.51)
nAA → AN	Weibull	Shape	0.43	(0.21, 0.77)
		Intercept	4.69	(2.98, 8.35)
		≥ 2 FDRs with CRC 50-70†	0.10	(-0.36, 0.62)
		≥ 2 FDRs with ≥ 1 CRC <50†	-0.21	(-0.80, 0.29)
		Sex:Male	0.32	(-0.13, 0.81)
		Age	0.0	(-0.03, 0.03)
		AA:Yes	**0.79**	**(0.12, 1.73)**
		Study:German§	0.42	(-0.19, 1.29)

nAA: Non advanced adenoma; **AN:** Advanced neoplasia; **HR:** Hazard ratio; **95% CrI:** 95% credible interval; **FDR:** first-degree relative.

† Reference group is 1 FDR with CRC < 50.

§ Included as an additional covariate in the model (i.e., a dummy variable) to account for possible cohort effect due to combining data from Dutch and German cohorts; similar to the approach by Lange et al. (2).

6 Additional results from the multivariate analysis based on the Dutch population only (i.e. sensitivity analysis)

To further assess the impact of differences in baseline characteristics between the Dutch and German cohorts, we repeated the analyses using only the Dutch cohorts. The analyses are consistent with those conducted for the total population (i.e., base-case analysis) as discussed in the main manuscript. Table S3 presents the parameter estimates of the full model, including the Weibull shape parameters, while Table S4 reports the HRs and corresponding 95% CrI for easier interpretation. The Weibull shape parameters for the progression times from HE to nAA and from nAA to AN were estimated to be 0.92 (95% Crl: 0.75–1.10) and 0.52 (95% Crl: 0.25–0.94), respectively. The former estimate, which is not statistically significant, suggests that the hazard of developing nAA from baseline could be constant over time. This contrasts with the analysis of the total population, where the corresponding estimate was statistically significant, indicating a decreasing hazard of developing nAA from baseline over time. The difference in statistical significance is likely due to the smaller sample size ($n = 734$ for the Dutch population and $n = 876$ for the total population). Additionally, the results of the analyses for the Dutch cohorts only were similar to those of the total population, with one exception. In contrast to the analysis of the total population (Table S2), none of the family history groups were statistically significantly associated with the hazard of developing nAA from baseline (Tables S3 and S4). Again, this difference in statistical significance could be due to the smaller sample size.

Table S3. Result of the multivariate analysis showing the parameter estimates of the full model with $n = 734$ individuals.

Transition	Distribution	Covariate	Estimate	95% Crl
Baseline → nAA	Weibull	Shape	0.92	(0.75, 1.10)
		Intercept	2.98	(2.70, 3.33)
		$\geq$ 2 FDRs with CRC 50-70†	0.16	(-0.12, 0.44)
		$\geq$ 2 FDRs with $\geq$ 1 CRC <50†	0.22	(-0.13, 0.57)
		Sex:Male	0.06	(-0.18, 0.31)
		Age	**0.04**	**(0.02, 0.05)**
		AA:Yes	0.44	(-0.08, 0.94)
nAA → AN	Weibull	Shape	0.52	(0.25, 0.94)
		Intercept	4.08	(2.54, 7.53)
		$\geq$ 2 FDRs with CRC 50-70†	0.14	(-0.40, 0.77)
		$\geq$ 2 FDRs with $\geq$ 1 CRC <50†	-0.34	(-1.26, 0.33)
		Sex:Male	0.18	(-0.33, 0.80)
		Age	0.01	(-0.03, 0.05)
		AA:Yes	**1.03**	**(0.22, 2.22)**

nAA: Non-advanced adenoma; **AA:** Advanced adenoma; **AN:** Advanced neoplasia; **HR:** Hazard ratio; **95% Crl:** 95% credible interval; **FDR:** First-degree relative.

† Reference group is 1 FDR with CRC $<$ 50.

Table S4. Hazard ratios showing the association between each covariate and the hazard of developing nAA from baseline and the hazard of developing AN since nAA onset in the Dutch population with 734 individuals.

**Transition**	**Distribution**	**Covariate**	**HR**	**95% CrI**
Baseline → nAA	Weibull	$\geq$ 2 FDRs with CRC 50-70[†]	1.17	(0.88, 1.57)
		$\geq$ 2 FDRs with $\geq$ 1 CRC <50[†]	1.25	(0.88, 1.77)
		Sex:Male	1.07	(0.83, 1.37)
		Age	**1.04**	**(1.02, 1.06)**
		AA:Yes	1.55	(0.92, 2.53)
nAA → AN	Weibull	$\geq$ 2 FDRs with CRC 50-70[†]	1.16	(0.66, 2.09)
		$\geq$ 2 FDRs with $\geq$ 1 CRC <50[†]	0.71	(0.27, 1.38)
		Sex:Male	1.19	(0.72, 2.20)
		Age	1.01	(0.97, 1.05)
		AA:Yes	**2.82**	**(1.24, 9.08)**

nAA: Non-advanced adenoma; **AA:** Advanced adenoma; **AN:** Advanced neoplasia; **HR:** Hazard ratio; **95% CrI:** 95% credible interval; **FDR:** First-degree relative.

[†] Reference group is 1 FDR with CRC $<$ 50.

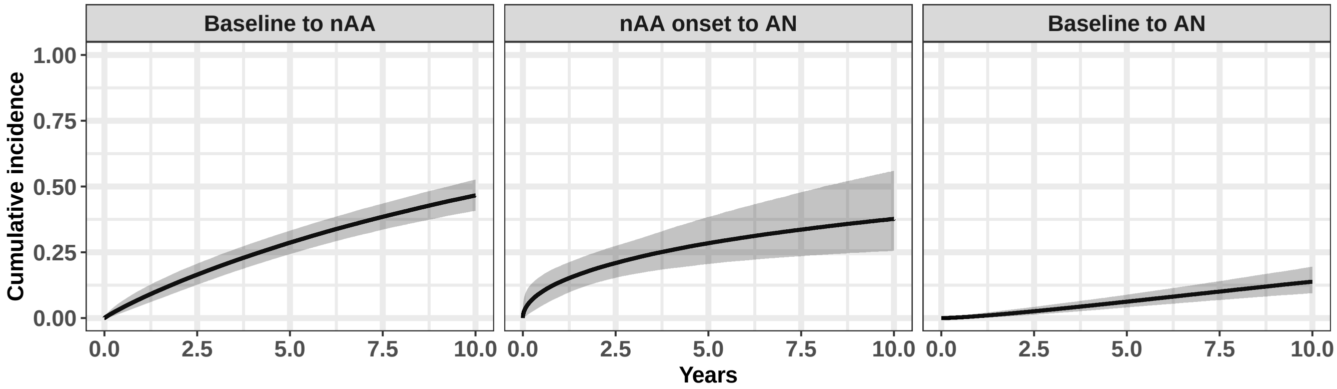

Figure S3. Estimated marginal cumulative incidence functions representing the population-average risks to develop nAA from baseline, AN after nAA onset and AN from baseline based on the Dutch population only. Estimates are based on the full multivariate model (Supplemental Table S3). The black solid line represents the average MCMC estimate while the grey shaded area represents the 95% CrI. Note that all individuals are included in the modelled curve, rather than depicting the cumulative incidence for a specific covariate profile.

A)

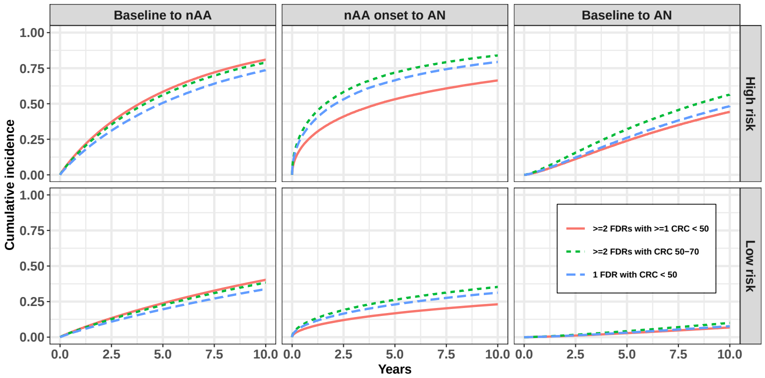

B)

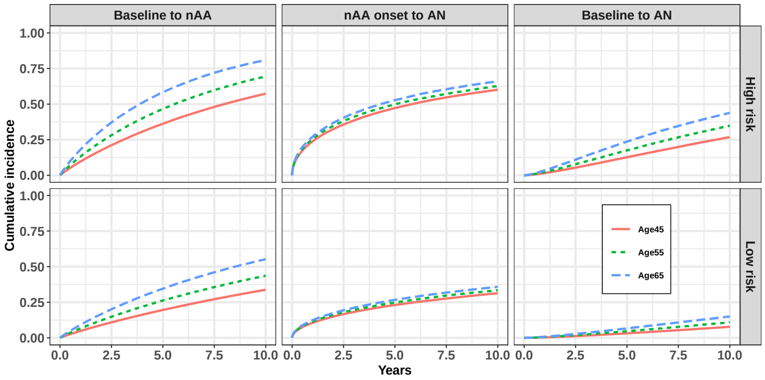

C)

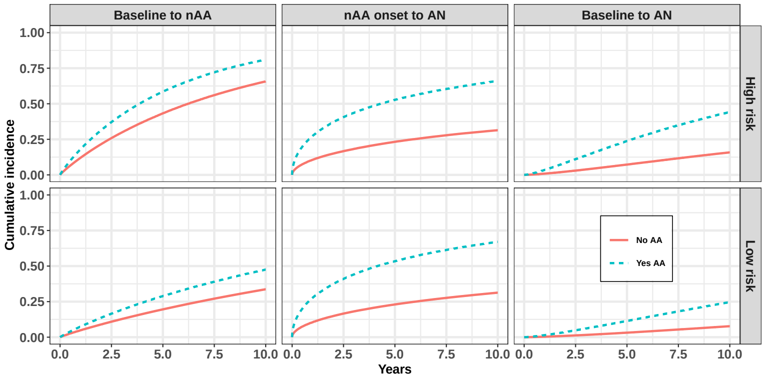

Figure S4. Conditional cumulative incidence functions representing the individual-specific risks to develop nAA from baseline, AN after nAA onset and AN from baseline based on the Dutch population only. Estimates for 'High-' and 'Low-risk' individuals were stratified by **(A)** the different family history groups; **(B)** different baseline ages (45, 55, 65 years); and the **(C)** presence of AA removed at baseline (yes/no). Note that estimates were based on the full multivariate model (Supplemental Table S3) and all other covariates were therefore based on a high- and low-risk profile. 'High-risk' individuals were defined as males aged 65 years with at least 2 FDRs diagnosed with CRC of which at least one was diagnosed before the age of 50. 'Low-risk' individuals were defined as females aged 45 years with 1 FDR who was diagnosed with CRC before age 50 years and with no AA removed at baseline.

7 Additional results comparing perfect sensitivity and imperfect sensitivity of colonoscopy in detecting nAAs (i.e. exploratory analysis)

This section presents additional results of the exploratory analysis. Figure S5 compares the marginal cumulative incidence function from a model assuming perfect (100%) sensitivity in detecting nAAs at all colonoscopies (both baseline and follow-up) to a model with 75% sensitivity for detecting nAAs during follow-up colonoscopies, but not for the baseline colonoscopy.

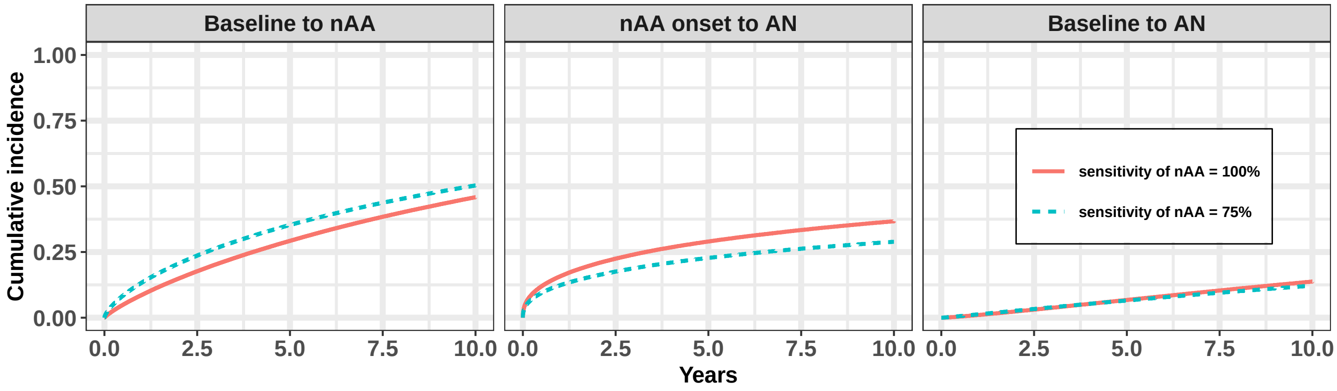

Figure S5. Marginal cumulative incidence functions representing the population-average risks to develop nAA from baseline, AN after nAA onset and AN from baseline based on the *total population*. The *red solid* and *blue dotted* lines represent 100% and 75% sensitivity in detecting nAAs, respectively. Note that all individuals in the population are included in the modelled curve, rather than depicting the cumulative incidence for a specific covariate profile.

References

1. Van Rijn JC, Reitsma JB, Stoker J, et al. Polyp miss rate determined by tandem colonoscopy: a systematic review. *Am J Gastroenterol.* 2006;101(2):343–350.

2. Lange JM, Gulati R, Leonardson AS, et al. Estimating and comparing cancer progression risks under varying surveillance protocols. *Ann Appl Stat.* 2018;12(3):1773.
